# Cardiac steatosis is associated with excess body weight in otherwise healthy adults

**DOI:** 10.1186/1532-429X-13-S1-O82

**Published:** 2011-02-02

**Authors:** Rajarshi Banerjee, Belen Rial, Joseph J Suttie, Pete Cox, Adam J Lewandowski, Andrew Johnson, Oliver Rider, Cameron Holloway, Jane Francis, Matthew D Robson, Jurgen Schneider, Kieran Clarke, Paul Leeson, Stefan Neubauer

**Affiliations:** 1 University of Oxford, Oxford, UK

## Background and aim

Obesity confers an enormous burden of cardiovascular morbidity and mortality worldwide. Lipotoxicity has been implicated as a potential common pathway in myocyte dysfunction and ultimately apoptosis. Fat deposition in the peritoneum, the liver and around vascular structures has been linked to metabolic syndrome and subsequent cardiomyopathy. Therefore this study sought to determine the relationship between excess body fat and intracardiac lipids as assessed by 3T proton spectroscopy in women.

## Methods

27 healthy female volunteers with no comorbidities were recruited from the general public according to body mass index - 14 lean (BMI 21.3 ± 2.0 kg/m^2^), 5 overweight (BMI 27.7 ± 1.7 kg/m^2^) and 8 obese (BMI 40.5 ± 8.1 kg/m^2^). All participants were scanned on a 3 Tesla magnet (Siemens Tim Trio). A stack of short axis cine images was obtained to measure cardiac volumes and mass. Cardiac proton spectroscopy was performed using a breath-hold STEAM sequence with water suppression on a septal mid-ventricular voxel. A transverse image at the level of the L4 vertebra was used to measure visceral and subcutaneous adipose tissue. All patients fasted for 10 hours prior to their study, and to ensure adequate hydration status water intake was encouraged.

## Results

**Figure 1:**
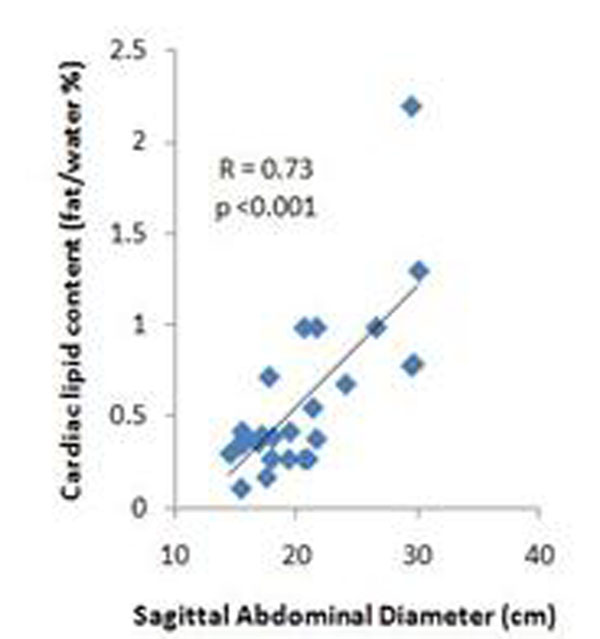
Cardiac lipid content increases with sagital abdominal diameter (cm).

**Figure 2:**
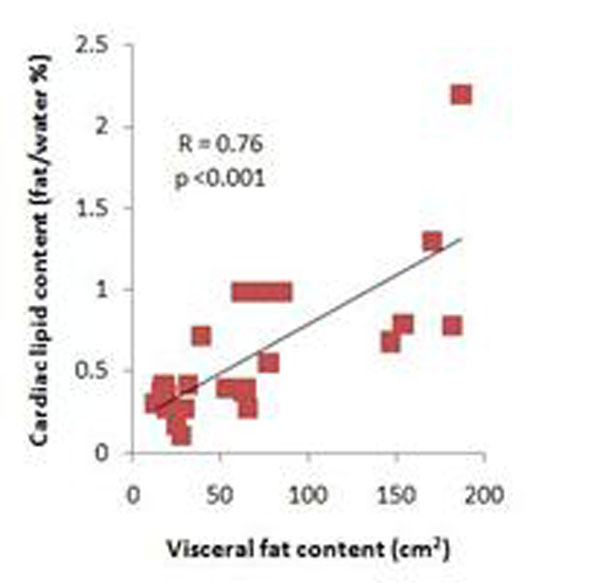
Intracardiac lipid content increases in associated with visceral fat content (cm^2^)

The normal range for intracardiac lipids in lean women was 0.36% +/- 0.14 of the total water signal, similar to previously reported ranges (1, 2). Cardiac lipid content was higher in overweight (0.55% +/- 0.31) and obese women (0.86% +/- 0.57; p = 0.01) compared to lean. Intracardiac lipid content correlated strongly with waist circumference, BMI and sagittal abdominal diameter (Fig 1). There was also a significant correlation between ICL and visceral adiposity (Fig 2) and total body fat (r = 0.74, p < 0.001). In this healthy population, there was no significant relationship between left ventricular ejection fraction and intracardiac lipids, but there was a significant correlation of lipid content with cardiac mass (r = 0.50, p = 0.01).

**Figure 3:**
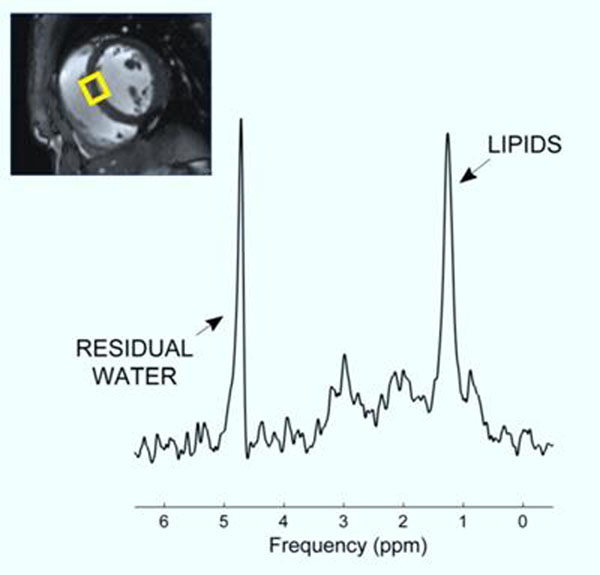


## Conclusions

Cardiac lipid content is increased in obese women even in the absence of diabetes and hyperlipidaemia. This may reflect increased lipid deposition within myocytes, and/or altered lipid usage & metabolism.

## References

[B1] McGavock Cardiac steatosis in diabetes mellitus: a 1H-magnetic resonance spectroscopy study Circulation 2007 116 10 1170 1175 10.1161/CIRCULATIONAHA.106.645614 17698735

[B2] Van der Meer Effects of short-term high-fat, high-energy diet on hepatic and myocardial triglyceride content in healthy men J Clin Endocrinol Metab 2008 93 7 2702 2708 10.1210/jc.2007-2524 18430773

